# Increasing gene discovery and coverage using RNA-seq of globin RNA reduced porcine blood samples

**DOI:** 10.1186/1471-2164-15-954

**Published:** 2014-11-04

**Authors:** Igseo Choi, Hua Bao, Arun Kommadath, Afshin Hosseini, Xu Sun, Yan Meng, Paul Stothard, Graham S Plastow, Christopher K Tuggle, James M Reecy, Eric Fritz-Waters, Samuel M Abrams, Joan K Lunney, Le Luo Guan

**Affiliations:** Animal Parasitic Diseases Laboratory, ARS, USDA, Beltsville, MD USA; Department of Agricultural, Food and Nutritional Science, University of Alberta, Edmonton, AB Canada; Department of Animal Science, Iowa State University, Ames, IA USA; Institute for Animal Science, Physiology and Hygiene Unit, University of Bonn, Katzenburgweg 7-9, 53115 Bonn, Germany

**Keywords:** Pig, Blood, Globin reduction, RNA-seq, Transcriptome

## Abstract

**Background:**

Transcriptome analysis of porcine whole blood has several applications, which include deciphering genetic mechanisms for host responses to viral infection and vaccination. The abundance of alpha- and beta-globin transcripts in blood, however, impedes the ability to cost-effectively detect transcripts of low abundance. Although protocols exist for reduction of globin transcripts from human and mouse/rat blood, preliminary work demonstrated these are not useful for porcine blood Globin Reduction (GR). Our objectives were to develop a porcine specific GR protocol and to evaluate the GR effects on gene discovery and sequence read coverage in RNA-sequencing (RNA-seq) experiments.

**Results:**

A GR protocol for porcine blood samples was developed using RNase H with antisense oligonucleotides specifically targeting porcine hemoglobin alpha (*HBA*) and beta (*HBB*) mRNAs. Whole blood samples (n = 12) collected in Tempus tubes were used for evaluating the efficacy and effects of GR on RNA-seq. The *HBA* and *HBB* mRNA transcripts comprised an average of 46.1% of the mapped reads in pre-GR samples, but those reads reduced to an average of 8.9% in post-GR samples. Differential gene expression analysis showed that the expression level of 11,046 genes were increased, whereas 34 genes, excluding *HBA* and *HBB*, showed decreased expression after GR (FDR <0.05). An additional 815 genes were detected only in post-GR samples.

**Conclusions:**

Our porcine specific GR primers and protocol minimize the number of reads of globin transcripts in whole blood samples and provides increased coverage as well as accuracy and reproducibility of transcriptome analysis. Increased detection of low abundance mRNAs will ensure that studies relying on transcriptome analyses do not miss information that may be vital to the success of the study.

**Electronic supplementary material:**

The online version of this article (doi:10.1186/1471-2164-15-954) contains supplementary material, which is available to authorized users.

## Background

Blood is a valuable resource to probe an animal’s physiological and pathological status as well as to obtain repeated samples before harvest, for example, monitoring the dynamic change of gene expression in response to disease, treatment, or aging, for which the onset of gene expression response is not known. However, transcriptomic analysis of blood samples is a challenge since blood is composed of heterogeneous cell types including red blood cells (99%), platelets (1%) and white blood cells (<1%; e.g., neutrophils, monocytes, basophils, lymphocytes and eosinophils) [1, 2]. In human blood, *HBA* and *HBB* are the most abundant transcripts (~52-76%) [3, 4]. The high level of globin transcripts in blood was reported to be the most limiting factor for accurate and sensitive detection of gene expression, especially for the less abundant transcripts [3–5]. This issue is a great concern for sequence-based approaches, in which the globin transcripts will be highly abundant and limit the potential coverage and detection of other transcripts from blood [3].

To date, several globin RNA reduction protocols have been successfully applied to gene expression studies in human [6–9]. GLOBINclear^TM^ (Ambion, Austin, TX, USA), a commercial product widely used in human clinical research, removes up to 95% of the *HBA* and *HBB* transcripts in human whole blood samples and improves the efficacy of gene expression assays [4, 10, 11]. Further approaches developed by Affymetrix (Affymetrix Inc., Santa Clara, CA, USA) [5, 11] or PNA Bio Inc. (Thousand Oaks, CA, USA) [9, 10] also have differential reduction rates of globin transcripts in human blood. Globin RNA reduction improved the sensitivity and reproducibility of high throughput mRNA expression analysis of whole human blood samples [3–5, 7, 9, 10]. There is, however, neither a commercial GR product available nor any literature demonstrating the efficiency and effects of GR at global level for porcine whole blood [2].

Our objectives were to develop a porcine specific GR protocol and to evaluate the effects of GR treatment on gene discovery and coverage in RNA-seq experiments for swine.

## Results and discussion

### Comparisons of globin reduction methods

To determine the suitability of the GR process for porcine whole blood samples, we initially evaluated the efficacies of three distinct methods (GLOBINclear^TM^, biotinylated PNA and RNase H) with whole blood samples drawn from 12 pigs collected in either PAXgene^TM^ (n = 3) or Tempus^TM^ (n = 9) tubes. To evaluate and compare GR efficiency, we performed qPCR analysis of *HBA* and *HBB* transcripts with a pooled sample for GLOBINclear and PNA methods and 5 randomly selected samples for the RNase H method (Additional file 1: Table S1). The GLOBINclear^TM^-Human Kit (Ambion, Austin, TX, USA), commonly used in human samples, seemed to have merit as it employs a non-enzymatic magnetic method but its reduction efficiency in pig barely reached 64% and 67% for *HBA* and *HBB* transcripts, respectively (Additional file 1: Figure S1). The manufacturer confirmed that porcine *HBA* and *HBB* sequences had low sequence homology to their corresponding human oligonucleotide probes used in the GLOBINclear^TM^-Human Kit, but the degree of dissimilarity is not known because the human probe sequences used in the GLOBINclear^TM^ Kit are not publicly available. Next, we designed porcine specific biotinylated PNA oligonucleotides and used them with the GLOBINclear^TM^ Kit. This PNA oligo method, however, reduced levels of *HBA* and *HBB* transcripts only 40% and 34%, respectively (Additional file 1: Figure S1). Third, we evaluated the RNase H mediated GR method using porcine specific oligonucleotides modified from the Affymetrix GeneChip GR Protocol developed for reduction of human globin transcripts [5]. We examined the sequence similarities of *HBA* and *HBB*, especially the oligonucleotide sequences on 3′ UTR, between human and pig using Clustal Omega (Additional file 1: Figure S2) [12]. Due to a lack of consensus, we designed two sets of porcine specific oligonucleotides each for *HBA* and *HBB* (Table 1). This revised RNase H mediated GR protocol resulted in an average reduction of 94% of *HBA* and 92% of *HBB* transcripts from porcine whole blood samples (Additional file 1: Figure S1). Thus we determined that the RNase H GR method using our custom designed porcine specific oligos was the most efficient of the three GR methods tested here and confirmed its efficacy by RNA-seq (Additional file 1: Table S2).

**Table 1:** **Porcine specific globin oligonucleotides used in RNase H-mediated globin reduction assay**

Gene	Sequence	Final Conc. (10X)
*HBA*	5′- GAT CTC CGA GGC TCC AGC TTA ACG GT -3′	7.5 uM
	5′- TCA ACG ATC AGG AGG TCA GGG TGC AA -3′	7.5 uM
*HBB*	5′- AGG GGA ACT TAG TGG TAC TTG TGG GC -3′	30 uM
	5′- GGT TCA GAG GAA AAA GGG CTC CTC CT -3′	30 uM

### Performance of GR protocol in an RNA-Seq experiment

After determining the valid and highly efficient GR method, we evaluated the effects of the RNase H GR treatment on gene discovery and coverage in an RNA-seq experiment. Since the above study on the GR method included samples collected using different blood collection tubes and RNA isolation methods, we evaluated the effects of the RNase H GR treatment on gene discovery and coverage in an RNA-seq experiment with a different set of 12 porcine blood samples collected in Tempus^TM^ tubes and for which the RNA was isolated by a magnetic bead based MagMax^TM^ kit.

**Figure 1:**
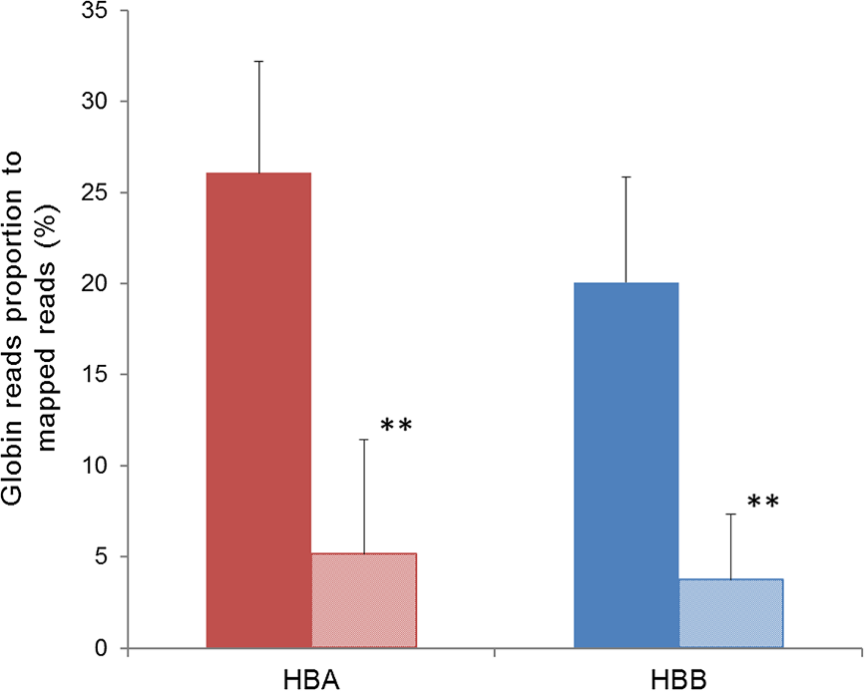
**The average proportions of** ***HBA*** **and** ***HBB*** **reads to total mapped reads in pre- and post-GR samples.** Solid and pattern bars show the average (± s.d.) proportions of globin reads in pre- and post-GR samples, respectively. RNase H mediated GR protocol decreased both proportions of *HBA* and *HBB* reads to mapped reads significantly (Paired Wilcoxon signed rank test, p <0.001).

More than 653 million (M) sequence reads generated from 12 pre- and post-GR samples passed Illumina’s CASAVA (v.1.8) filtration (Table 2). These reads were then aligned to the pig genome build 10.2 by TopHat (v. 2.0.8). After GR treatment, total filtered reads and mapped reads were reduced by an average of 6.1 M and 6.8 M reads, respectively, and globin reads were reduced by an average of 11.4 M reads. The percentage of globin reads among all aligned reads averaged 46.1% and of these, 84.7% were removed by GR treatment. The proportion of globin reads to mapped reads were 46.1% and 8.9% in pre- and post-GR samples, respectively, and proportions of *HBA* and *HBB* reads to mapped reads were significantly reduced to 5.2% from 26.1% and to 3.7% from 20.1%, respectively (p <0.001, Figure 1). Considering that human globin transcripts constitute 50-70% of the blood RNA [3, 4], the level of pig globin transcripts in pre-GR samples is comparatively low. A possible explanation for the lower level of porcine globin transcripts is that the pigs used in this study were only 1-2 months old, an age associated with rapid decreases in erythrocyte population size and hemoglobin concentration. Although pigs at birth have similar hematological values to adult pigs, by three days of age a 25% reduction in hemoglobin concentration has occurred and hemoglobin concentration then increased gradually from the age of 3 months due to the pig’s tremendous early growth rate, as much as eight times faster than humans [13, 14]. Thus, we expect the GR protocol will reduce more globin transcripts in newborn and adult pig blood RNAs.

**Table 2:** **RNA-seq mapping statistics for pre- and post-globin reduction samples**

	pre-Globin Reduction						post-Globin Reduction					
Sample	RIN ^a^	Total reads ^b^	Aligned reads ^b^	Expressed genes ^c^	HBA reads ^b^	HBB reads ^b^	RIN ^a^	Total reads ^b^	Aligned reads ^b^	Expressed genes ^c^	HBA reads ^b^	HBB reads ^b^
1	7.8	23.66	22.45	11269	7.57	4.19	8.0	18.00	16.45	12175	2.35	1.60
2	7.7	19.73	18.57	11591	5.43	3.40	7.8	21.62	19.56	12736	2.16	1.29
3	7.6	26.12	24.62	11445	8.24	4.96	7.7	21.68	19.91	12246	3.69	2.17
4	7.9	23.78	22.29	11796	6.68	3.80	7.5	25.57	22.94	12942	1.93	1.28
5	7.5	23.67	22.38	10966	7.23	4.63	7.4	19.81	17.59	12409	0.29	0.26
6	6.8	24.58	22.79	10671	6.30	3.82	7.0	17.79	15.60	11613	0.26	0.17
7	6.8	23.53	21.42	11517	2.82	2.76	6.4	21.03	18.50	11736	0.34	0.20
8	6.5	22.01	20.18	11900	4.54	2.13	5.9	18.40	16.20	12203	0.08	0.06
9	5.0	45.16	41.89	11464	9.36	12.64	4.9	25.33	21.86	11834	0.28	0.55
10	4.7	39.39	36.09	11319	8.46	8.37	4.5	34.33	29.36	12214	0.17	0.27
11	4.2	46.71	42.13	11292	8.92	9.74	4.1	34.64	29.45	11680	0.31	0.74
12	3.7	44.94	41.68	10441	9.96	12.16	3.5	31.81	27.31	11322	0.30	0.48

^a^RIN: RNA Integrity Number provided by Agilent 2100 bioanalyzer.

^b^Paired read counts are given in millions.

^c^Genes are considered expressed when read count >5.

A total of 24 samples were loaded onto different lanes according to RIN measured after globin reduction (sample 1–6: high RIN, sample 7–8: moderate RIN and sample 9–12: low RIN).

### Classification of samples based on RNA integrity number (RIN)

We examined the RIN changes after GR treatment of pig blood RNA and its effect on sequencing results. The quality of RNA was not changed overall (p >0.1); though 8 samples showed a reduction in RIN after GR treatment, only 3 samples showed a marked decrease of RIN (0.4-0.6) after GR. However, there was a reduction in RNA yield following GR treatment with only 33.3-78.2% of total RNA being recovered. Studies on GR treatment in humans also reported the reduction of RNA yields ranging from 52-95% of total RNA [3, 4, 7, 15]. The reasons for the significant reduction and the wide variation in RNA yield are not clear. To offset the RNA loss accompanying GR treatment, it would be desirable to prepare sufficient amounts of initial RNA. Because we identified possible bias introduced by RIN from the preliminary sequencing results (data not shown), we empirically classified the samples into three categories based on RIN after GR treatment: high (RIN ≥7), moderate (5 ≤ RIN <7), and low (RIN <5) representing ideal, critical and inferior RNA integrity for RNA-seq experiments, respectively.

### Increased coverage of non-globin genes in post-GR samples

**Figure 2:**
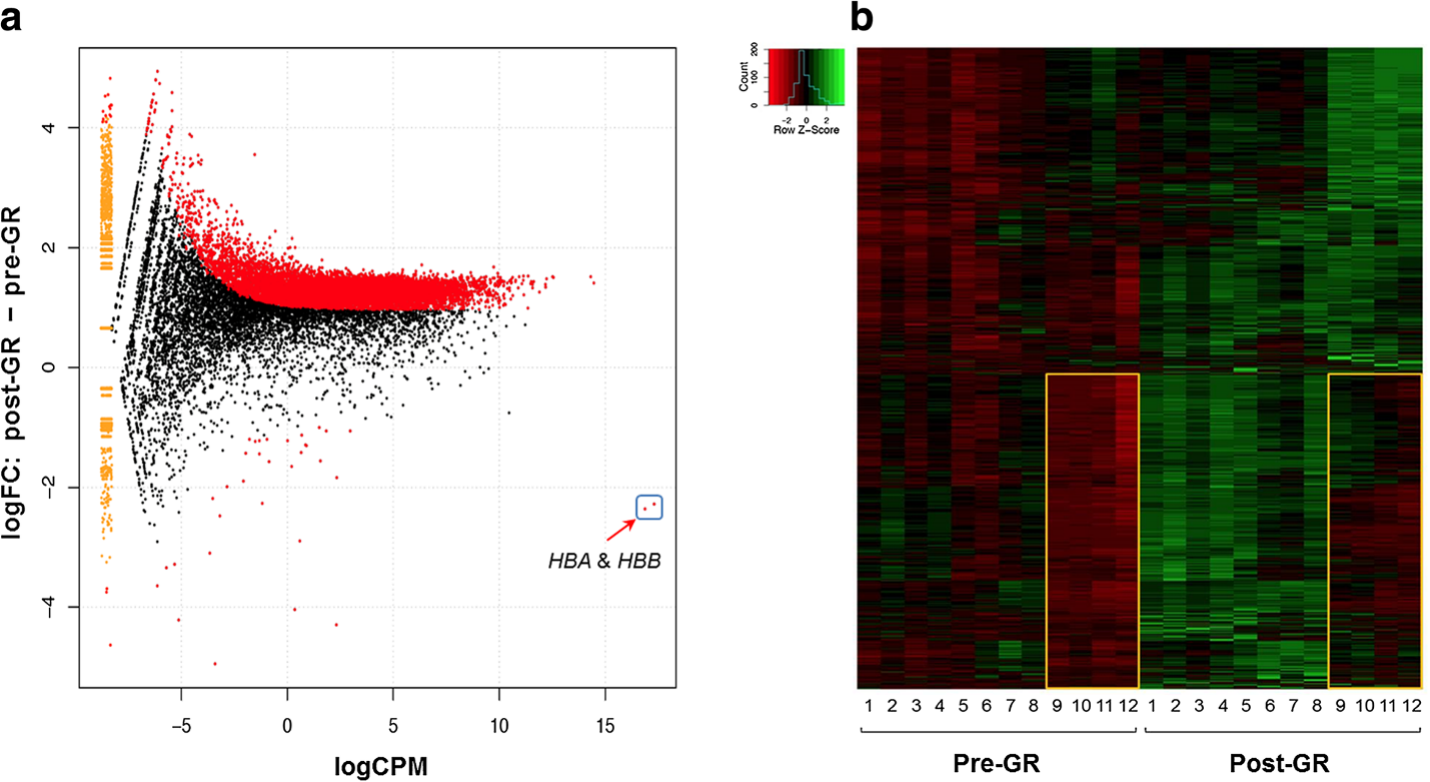
**Differential gene expression in pre- versus post-GR samples. a**. MA plot revealed that the majority of the differentially expressed genes showed increased detection (11,046) in post-GR samples and only 34 (excluding globin genes) showed lower detection. **b**. Heatmap shows the expression profile across all samples for the 11,046 genes with increased detection by GR treatment. A large set of genes in the low RIN samples (within the orange boxes) was considerably lower expressed than the corresponding set in the high/moderate RIN samples.

Following an approach similar to that described by Mastrokolias *et al*. [3], we investigated the effect of GR on enhancing the coverage of non-globin genes and the sensitivity of gene expression detection. Read count data was normalized by library size and DE genes between pre- and post-GR samples were determined using edgeR (see Methods). Compared to pre-GR samples, 11,046 genes showed higher level of detection (expression) and 34 genes (Table 3), excluding *HBA* and *HBB* and ENSSSCG00000014727 (hemoglobin subunit beta-like), showed lower level of detection after GR treatment (FDR <0.05) (Figure 2a). We checked for sequence similarities among these 34 genes and the four globin oligonucleotides for possible non-target specific hybridization, but found none.

**Figure 3:**
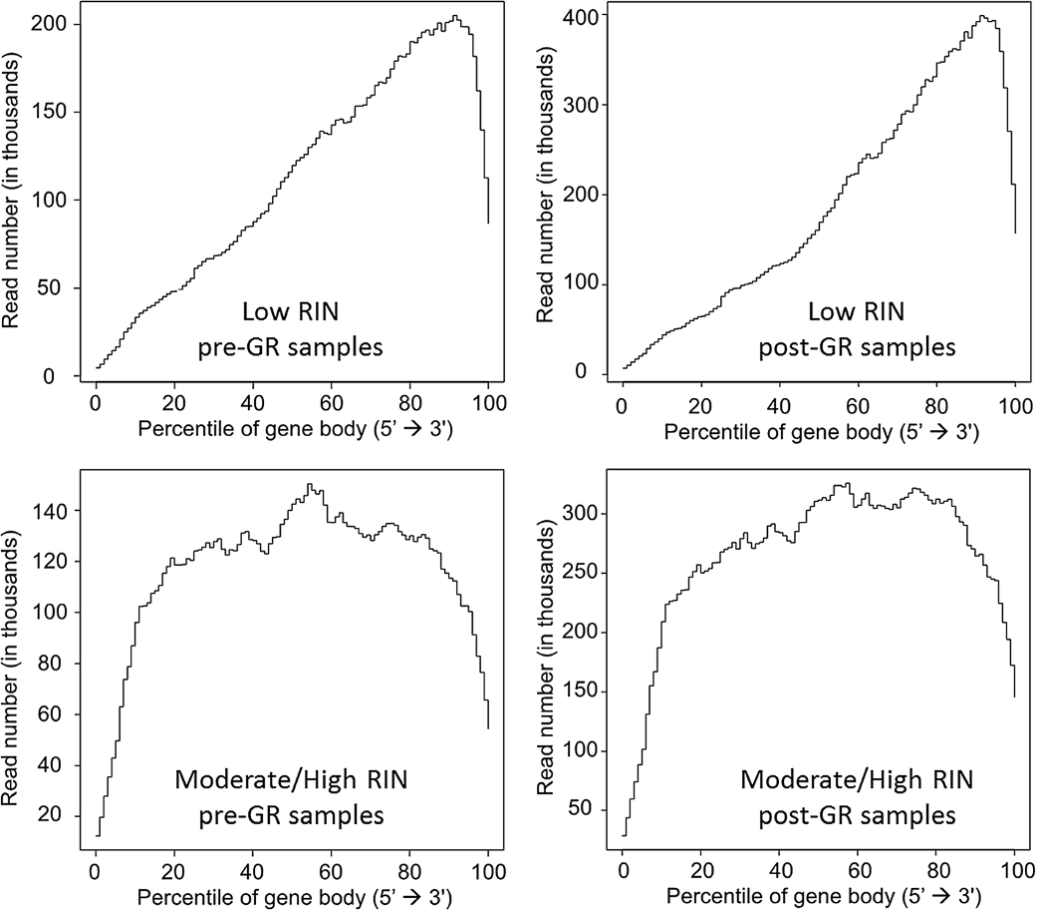
**Normalized 5′ to 3′ sequence coverage by position showing lower expression in one representative low RIN sample.** For lower expressed 4,792 genes in low RIN samples, the average read numbers of low RIN (<5) and moderate/high RIN (≥5) in pre- and post-GR samples is shown at each relative position.

Figure 2b depicts a heatmap of the normalized log2 transformed expression of the 11,046 genes with higher level of detection in post-GR samples compared to pre-GR samples. It was observed that a large set of genes in the low RIN samples (within the boxes in Figure 2b) was considerably lower expressed than the corresponding set in the high/moderate RIN samples, both pre- and post-GR. We believe that these are the genes with the greatest degradation in the low RIN samples. We then examined the variation in gene body coverage from 5′ to 3′ in high/moderate and low RIN samples, respectively. Low RIN samples showed strong bias toward increased coverage at the 3′ end (Figure 3). Among the low-quality RNA samples, pre- and post-GR treatment showed the same trend of bias, which affirmed that the RNase H treatment was not the determining factor. High quality samples showed better coverage from 5′ to 3′ as well as at the ends in both pre- and post-GR treated samples. All low quality samples were biased toward increased coverage at the 3′ end, possibly due to the degradation of RNA. However, the number of unique genes detected did not differ significantly between low and high RIN samples.

**Table 3:** **The list of 34 genes that showed decreased expression level after globin reduction**

Ensembl ID	Gene ^a^	Gene description ^a^
ENSSSCG00000028278	*POLR2A*	polymerase (RNA) II (DNA directed) polypeptide A, 220 kDa
ENSSSCG00000022476	–	–
ENSSSCG00000012570	–	–
ENSSSCG00000024020	*RERE*	arginine-glutamic acid dipeptide (RE) repeats
ENSSSCG00000028814	*SOD3*	superoxide dismutase 3, extracellular
ENSSSCG00000031032	*SLA-DRB3* ^b^	MHC class II, DR beta-like 3 pseudogene
ENSSSCG00000006328	*RXRG*	Retinoic acid receptor RXR-gamma
ENSSSCG00000003562	*SFN*	stratifin
ENSSSCG00000004389	–	–
ENSSSCG00000000386	*ANKRD52*	ankyrin repeat domain 52
ENSSSCG00000011331	–	–
ENSSSCG00000010913	–	–
ENSSSCG00000022894	*GZMH*	granzyme H
ENSSSCG00000027961	–	–
ENSSSCG00000011258	*EXOG*	endo/exonuclease (5′-3′), endonuclease G-like
ENSSSCG00000022933	*EFR3B*	EFR3 homolog B (S. cerevisiae) [Source: HGNC Symbol; Acc:29155]
ENSSSCG00000029574	–	–
ENSSSCG00000010915	–	–
ENSSSCG00000022681	–	–
ENSSSCG00000000253	*KRT18*	keratin 18
ENSSSCG00000029399	–	–
ENSSSCG00000013264	*PHF21A*	PHD finger protein 21A
ENSSSCG00000009603	–	–
ENSSSCG00000009444	*OLFM4*	olfactomedin 4
ENSSSCG00000030191	–	–
ENSSSCG00000029430	–	–
ENSSSCG00000023324	*KMT2A*	lysine (K)-specific methyltransferase 2A
ENSSSCG00000015124	*PVRL1*	poliovirus receptor-related 1 (herpesvirus entry mediator C)
ENSSSCG00000025712	–	–
ENSSSCG00000013547	*CRB3*	crumbs homolog 3 (Drosophila)
ENSSSCG00000026303	*PROSER1*	proline and serine rich 1
ENSSSCG00000003942	–	–
ENSSSCG00000013545	*TUBB4A*	tubulin, beta 4A class IVa
ENSSSCG00000001513	*SYNGAP1*	synaptic Ras GTPase activating protein 1

^a^Orthologous human gene symbol provided where available. Unannotated genes was presented as “–”.

^b^pseudogene.

The lower detection levels of a small number of genes in post-GR samples could also be due to the effect of RIN. We investigated all genes with decreased level of detection after GR (fold change <0) from each sample independently, regardless of statistical significance (Additional file 1: Figure S3). We observed that samples with the most notable RIN change after GR (RIN reduction ≥0.4) had the highest number of genes with decreased expression level (samples 4, 7 and 8; Additional file 1: Figure S3). In addition to the effect of RIN, technical variations or sampling effects could also contribute to differences in detection levels of genes.

### Increased number of non-globin genes identified in post-GR samples

**Figure 4:**
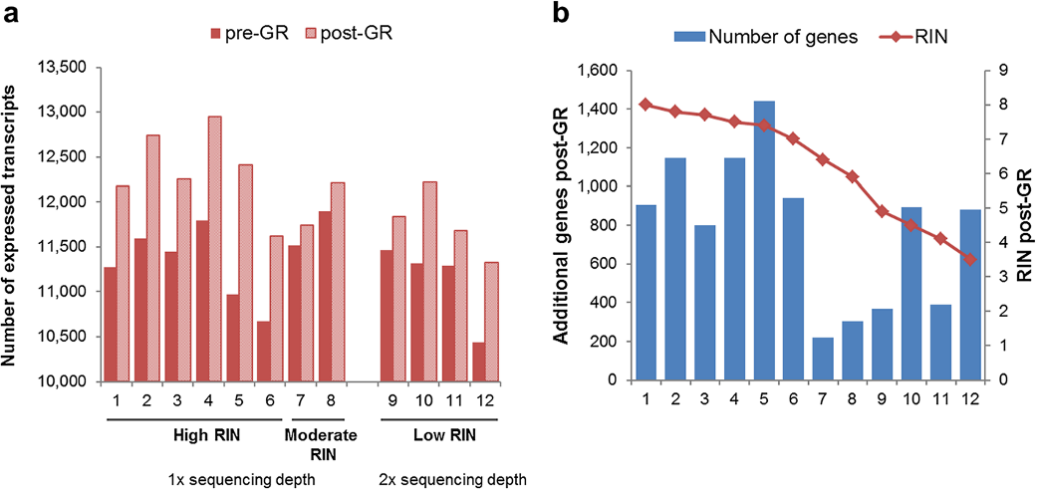
**Increased gene coverage as a result of globin reduction.** GR treatment increased the detection of expressed genes (read >5). 6 high (RIN ≥7) and 2 moderate (5 ≤ RIN <7) RIN samples were sequenced in one lane and 4 low (RIN <5) RIN samples were sequenced in another lane. **a)** Comparisons of number of expressed genes in pre- and post-GR treatment. **b)** RIN influence on identifying additional genes post-GR treatment.

The number of detected genes (read counts >5) in post-GR samples was significantly increased compared to pre-GR samples (paired t-test) (Figure 4a). GR treatment increased the gene detection rate by 8.6% in high RIN samples, 2.2% in moderate and 5.4% in low RIN samples. It was also noticed that the number of additional genes identified in post-GR samples was higher for samples with a high RIN (Figure 4b). It may be noted that the detection rate was higher in high RIN samples compared to low RIN samples despite being sequenced at half the depth. Pre-GR, an average of 93 genes were uniquely detected in the high RIN group, whereas 243 genes were uniquely detected in the moderate/low RIN group. Post-GR, the corresponding uniquely detected genes in the two groups were 1,157 and 753, respectively (Additional file 1: Figure S4).

**Figure 5:**
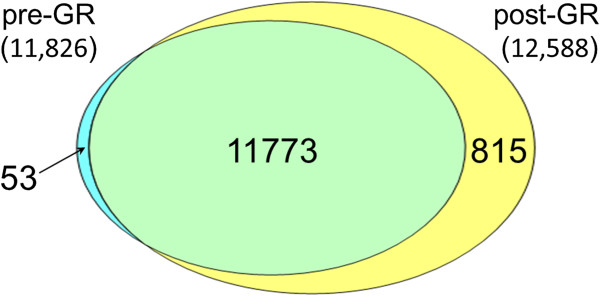
**Venn diagram of number of genes identified as expressed in pre- versus post-GR samples.** There is significant overlap (11,773) between pre- and post-GR samples. An additional 815 genes were identified in only post-GR samples.

We next determined genes expressed in porcine whole blood using all 12 samples, based on the criterion that a gene was detected at read counts above 5 in at least 5 of the 12 samples. We identified 12,588 genes in post-GR samples and 11,826 genes in pre-GR samples with an overlap of 11,773 genes (Figure 5). Excluding the overlap, 815 genes were detected only in post-GR samples, whereas 53 were specific to pre-GR samples. The small number of genes found specific to pre-GR samples may be due to the effect of RIN or technical variations. A comparison of the mean expressions of the set of 11,773 genes detected in both pre- and post-GR samples and the 815 genes detected only in post-GR samples revealed increased expression in post-GR samples (Additional file 1: Figure S5). The mean expression of the 815 additional genes in post-GR samples was well below the lower quartile of the expression levels of genes common to both pre- and post-GR samples. Thus GR treatment increases the ability to detect genes expressed at very low levels.

## Conclusions

The porcine specific GR protocol described here successfully removed a significant proportion of the *HBA* and *HBB* transcripts prior to sequence analysis. The range of gene discovery from RNA sequencing was extended with significant increases in number of identified genes via improved coverage. Our DE analyses using the GR samples showed increased sensitivity, with no observed strong negative effects as a result of the GR protocol. We also demonstrated the effects of RIN on blood RNA-seq analyses. Thus, the GR protocol incorporated into porcine blood transcriptomics will help advance pig physiological, pathological and blood biomarker studies, by providing more targets for drug development and disease resistance research.

## Methods

### Blood samples and RNA isolation

Animal protocols were approved by the Kansas State University and University of Alberta Animal Care and Use Committees. A total of 24 blood samples were used to conduct two independent studies: comparisons of three GR methods to select the best method and evaluating the effects of the selected GR method on an RNA-seq experiment. For the first study, 3 mL of blood samples from 9 pigs of 1-2 months age produced from Landrace x Large White selected from a commercial populations used in the Porcine reproductive and respiratory syndrome Host Genetics Consortium (PHGC) studies [16] were collected in Tempus^TM^ Blood RNA tubes (Life Technologies, Carlsbad, CA, USA) and 2.5 mL of blood samples from 3 crossbred pigs of Large White x Landrace were collected in PAXgene^TM^ Blood RNA tubes (PreAnalytiX, Qiagen, Germany) at the University of Alberta. Total RNA was isolated with PAXgene^TM^ Blood RNA Kit (PreAnalytiX, Qiagen) for PAXgene^TM^ tubes and either Tempus^TM^ Spin RNA Isolation Kit (Life Technologies, Carlsbad, CA, USA) or magnetic bead based MagMax^TM^ for Stabilized Blood Tubes RNA Isolation Kit for Tempus tubes (Life Technologies), according to the respective manufacturer’s instructions.

For the second study evaluating the effects of RNase H mediated GR protocol on RNA-seq, another set of 12 blood samples were drawn from crossbred pigs of Duroc x (Landrace x Yorkshire) in a PHGC population. Three mL of blood from each pig at 1-2 months of age was collected into Tempus^TM^ Blood RNA Tubes at Kansas State University. Total RNA was isolated using the MagMax^TM^ for Stabilized Blood Tubes RNA isolation kit according to the manufacturer’s protocol.

RNA concentration was quantified using a NanoDrop ND-1000 spectrophotometer (Nano-Drop Technologies, Wilmington, DE, USA) and RNA quality was assessed using an Agilent Bioanalyzer 2100 (Agilent Technologies, Inc., Santa Clara, CA, USA). To determine an accurate 28S/18S rRNA ratio in the pig, we aligned the human 28S sequence against pig genome build 10.2 using BLAST and identified 97-100% of similarity on pig chromosome 6: 871128-866484 (Ensembl release 73). The sizes of the 28S and 18S genes in pig were estimated to be 4645 bp and 2302 bp, respectively, yielding an rRNA ratio of 2.02, whereas the rRNA ratios in human and mouse are known to be 2.69 and 2.53, respectively (the ratio obtained from Genbank database; M11167 and X03205 in human and NR003279 and NR003278 in mouse).

### Design of porcine specific oligonucleotides

We first tested GLOBINclear^TM^ Human Kit (Ambion, Austin, TX, USA) which hybridized biotinylated oligonucleotides with globin transcripts by binding to Streptavidin Magnetic Beads. Second, we designed porcine specific biotinylated Peptide Nucleic Acids (PNA) oligos to inhibit reverse transcription of globin transcripts (*HBA*: 5′-CGAGGCTCCAGCTTA-3′ and *HBB*: 5′-CACCAGCCACCACCT-3′). Third, we designed four porcine specific antisense oligonucleotides for *HBA* and *HBB* using Primer 3 (v. 0.4.0) (Table 1) to hybridize with globin transcripts prior to digestion with RNase H. To design porcine specific oligonucleotides, we first used Clustal Omega (http://www.ebi.ac.uk/Tools/msa/clustalo/) to align the porcine *HBA* (ENSSSCT00000008741) and *HBB* (ENSSSCT00000016076) transcript sequences in the current assembly of the pig genome (build 10.2) with their orthologues from human, mouse, cow and pig obtained from the Ensembl database (http://www.ensembl.org) and then checked the similarity of the 3′ end hybridization sites (Additional file 1: Figure S2).

### Globin reduction treatment

GR treatment with porcine specific oligonucleotides was performed using a modified Affymetrix GR protocol [5]. In brief, 10X GR oligonucleotides mix was prepared adding 100 uL each of two *HBA* Oligos at 30 uM, two *HBB* Oligos at 120 uM per reaction, yielding a final concentration of 7.5 uM *HBA* Oligos and 30 uM *HBB* Oligos. Three ug of denatured total RNA was hybridized in a thermal cycler at 70°C for 2 min with the 400 uL 10X GR oligonucleotides mix in hybridization buffer (100 mM Tris-HCL, pH 7.6; 200 mM KCl) at 70°C for 5 min, then cooled to 4°C. The RNA-DNA hybrids were digested with 2 U RNase H (Ambion) in the reaction buffer (100 mM Tris–HCl, pH 7.6, 20 mM MgCl_2_, 0.1 mM DTT, SUPERase-In) at 37°C for 10 min and cooled to 4°C. The reaction was stopped by addition of 1.0 ul 0.5 M EDTA. RNase H treated RNA was immediately purified with the RNeasy MinElute Cleanup Kit (Qiagen, Toronto, Canada, Cat. No.: 74204) according to manufacturer’s instructions. RNA quality of GR treated samples was assessed using an Agilent Bioanalyzer 2100 (Agilent Technologies, Inc.).

### Quantitative real-time PCR (qPCR) analysis

We quantified the mRNA level of the porcine *HBA* and *HBB* transcripts by SYBR Green I based qPCR using a StepOne^TM^ Real-Time PCR System (Applied Biosystems, Foster City, CA, USA). First strand cDNA was synthesized using SuperScript® II reverse transcriptase (Invitrogen) and random hexamer primers in a final volume of 20 μL following the manufacturer’s instruction. SYBR Green I based qPCR was performed in a total volume of 10 μL per reaction comprising 2 μL of template, 1 μL of the assay-specific primer mix, 5 μL of the Fast SYBR® Green Master Mix Bulk Pack (Applied Biosystems) and 2 μL of water. The reaction conditions used were one cycle of 95°C for 3 min for initial denaturation, 23 cycles of 95°C for 30 s and 60°C for 30 s. The primer sequences are shown in Additional file 1: Table S1.

### Library preparation for sequencing

Poly-A + fractions from the GR treated samples and respective non GR treated samples (1.5 μg RNA each) were purified by using oligo-dT magnetic beads (Illumina, Inc., San Diego, USA), and used to construct cDNA libraries. The Poly (A+) RNA was primed with random hexamers and fragmented at 94°C for 8 min. Second strand cDNA was synthesized after the first strand cDNA using SuperScript II (Invitrogen). The cDNA fragments were end-repaired and a single ‘A’ nucleotide was added to 3′-ends to prevent them from cross ligation during the adapter ligation step. Then individual RNA adapter index oligos were ligated to the end-repaired cDNA and subsequently amplified using Veriti Thermo cycler (Applied Biosystems). The initial denaturation was performed at 98°C for 30 seconds, followed by 15 cycles at 98°C for 10 seconds, annealing at 60°C for 30 seconds and extension at 72°C for 30 seconds. The final extension was followed at 72°C for 5 minutes, and held at 10°C.

The quality and size (~260 bp) of the resulting cDNA libraries were assessed using the High sensitivity DNA Kit (Agilent Technologies, Inc.) in an Agilent Bioanalyzer 2100 (Agilent Technologies, Inc.). The quantification was performed using StepOne^TM^ Real-Time PCR System (Applied Biosystems), as suggested in the Sequencing Library qRT-PCR Quantification Guide (Illumina, Inc.). The KAPA SYBR® FAST ABI Prism qPCR Kit (Kapa Biosystems, Inc., Woburn, USA) was used for the qPCR reactions. The individual libraries were pooled into 2 nM after quantification.

Sequencing was performed on the HiSeq System (Illumina, Inc.). The pooled 10 μL of the 2 nM libraries were diluted and denatured. The pooled cDNA libraries (12 pM) were loaded on the cBot (Illumina, Inc.) for clustering on a flow cell, and single-read cluster generation proceeded using the TruSeq^TM^ SR Cluster Generation Kit v3 (Illumina, Inc., Cat.: FC-930-3001). A portion of each library was diluted to 10 nM and stored at -20°C. Fifty cycles of sequencing-by-synthesis using the paired-end protocol was performed on a HiSeq (Illumina, Inc.) according to manufacturer’s instructions. Real-time analysis and base calling was performed using the HiSeq Control Software Version 1.4.8 (Illumina, Inc.).

### Bioinformatic analysis

Sequence reads with base quality scores were produced by the Illumina sequencer. Raw reads were processed using the Illumina CASAVA (v. 1.8) to filter out the low-quality reads. Sequence reads were then aligned to the pig genome reference assembly (build 10.2; [17]) using TopHat 2.0.8 [18] with default parameters. The number of reads uniquely mapped to each gene (Ensembl 71 annotation) was determined using Htseq-count (v0.5.3.p3; [19]). To determine number of genes identified in each sample, we required a read count >5.

To identify genes detected at decreased or increased levels between the globin reduced and non-reduced samples, the read count data were analysed using edgeR (version 3.0.8) [20] in R (version 2.15.2), as described [3]. Count data was normalized by the library size to account for different numbers of reads obtained from each sample. To determine differences in detection levels between the two groups, an exact test for the negative binomial distribution was used. The genes were considered to be differentially detected at FDR <0.05. RSeQC (v2.3.3) [21] was used for read distribution over gene body to check 5′/3′ bias. We used BlastN (v2.2.25) [22] to perform the alignment between globin oligos and the genes with decreased levels after GR treatment.

## Electronic supplementary material

Additional file 1: Table S1.: Primer sequences used in qPCR. **Table S2.** Blood collection tube, RNA isolation methods, sequence statistics, number of expressed genes and globin reads count in pre- and post-globin reduction samples. **Figure S1.** qPCR results for *HBA* and *HBB* gene expression comparing three different globin reduction methods. **Figure S2.** Alignment of orthologous *HBA* and *HBB* cDNA sequences in human, mouse, cattle and pig. **Figure S3.** Differential gene expression in pre- and post-GR samples. **Figure S4.** Individual Venn diagrams showing the number of genes detected by RNA-seq in pre- and post-GR samples. **Figure S5.** Comparison of the mean expressions of the set of genes detected in both pre- and post-GR samples and the genes detected only in post-GR samples. (DOCX 570 KB)
