# Supplementary material for: Increasing gene discovery and coverage using RNA-seq of globin RNA reduced porcine blood samples
Source: BMC Genomics. 2014 Nov 4;15(1):954. doi: 10.1186/1471-2164-15-954 (PMC4230834; doi:10.1186/1471-2164-15-954)
Supplement: Supplementary file 1 — Additional file 1: Table S1.: Primer sequences used in qPCR. Table S2. Blood collection tube, RNA isolation methods, sequence statistics, number of expressed genes and globin reads count in pre- and post-globin reduction samples. Figure S1. qPCR results for HBA and HBB gene expression comparing three different globin reduction methods. Figure S2. Alignment of orthologous HBA and HBB cDNA sequences in human, mouse, cattle and pig. Figure S3. Differential gene expression in pre- and post-GR samples. Figure S4. Individual Venn diagrams showing the number of genes detected by RNA-seq in pre- and post-GR samples. Figure S5. Comparison of the mean expressions of the set of genes detected in both pre- and post-GR samples and the genes detected only in post-GR samples. (DOCX 570 KB) [file 12864_2014_6644_MOESM1_ESM.docx]

**Table S1. Primer sequences used in qPCR**

| Gene | Sequence (5′ - 3′) |
| --- | --- |
| *HBA* | CCC ACC ACC CCG ATG ATT TC |
|  | TCA ACG ATC AGG AGG TCA GG |
|  |  |
| *HBB* | CTC CTG GGC AAC GTG ATA GT |
|  | GGT TCA GAG GAA AAA GGG CTC CTC CT |
| Reference gene | |
| *GAPDH* | CTT CAC GAC CAT GGA GAA GG |
|  | CCA AGC AGT TGG TGG TAC AG |
| *RPL19* | CCA GTG TCC TCC GCT GTG GC |
|  | CGG GAG TTG GCG TTG GCG AT |

**Table S2. Blood collection tube, RNA isolation methods, sequence statistics, number of expressed genes and globin reads count in pre- and post-globin reduction samples.**

| Blood  collection  tube | RNA Isolation |  | pre-Globin Reduction | | | | |  | post-Globin Reduction | | | | |
| --- | --- | --- | --- | --- | --- | --- | --- | --- | --- | --- | --- | --- | --- |
|  |  | Sample | Total  reads^a^ | Aligned  reads^a^ | Expressed genes^b^ | HBA  reads^a^ | HBB  reads^a^ |  | Total  reads^a^ | Aligned  reads^a^ | Expressed genes^b^ | HBA  reads^a^ | HBB  reads^a^ |
| PAXgene | Spin Column | P1 | 49.7 | 38.1 | 12886 | 4.3 | 3.3 |  | 41.2 | 31.5 | 11859 | 0.06 | 0.07 |
|  |  | P2 | 46.5 | 34 | 12149 | 6 | 7.1 |  | 31 | 24 | 12353 | 1.68 | 0.53 |
|  |  | P3 | 38.4 | 29.3 | 12775 | 3.8 | 2.9 |  | 46 | 31.9 | 12567 | 0.8 | 0.6 |
| Tempus | Spin Column | P4 | 37.6 | 29.8 | 12492 | 8.6 | 3.7 |  | 36.8 | 29.6 | 12162 | 0.07 | 0.03 |
|  |  | P5 | 38.2 | 30.3 | 12578 | 7.6 | 3.6 |  | 36.9 | 29.8 | 12178 | 0.09 | 0.01 |
|  |  | P6 | 36.5 | 28.5 | 11833 | 9.8 | 6 |  | 22 | 17.6 | 10890 | 0.12 | 0.01 |
|  |  | P7 | 58.5 | 45.7 | 12631 | 10 | 5.7 |  | 46.5 | 36.8 | 12498 | 2.4 | 0.8 |
|  |  | P8 | 70 | 53.2 | 12918 | 13.8 | 11 |  | 50 | 39.4 | 12853 | 6.3 | 3.5 |
|  | Magnetic bead | P9 | 13 | 10 | 6657 | 5 | 3 |  | 31 | 24 | 10367 | 0.09 | 0.77 |
|  |  | P10 | 28 | 22 | 8853 | 11 | 6.5 |  | 22 | 17 | 10385 | 0.12 | 0.6 |
|  |  | P11 | 20 | 16 | 10730 | 5.5 | 4.6 |  | 17 | 13 | 10951 | 0.02 | 0.4 |
|  |  | P12 | 22 | 17 | 11210 | 6.4 | 4.1 |  | 27 | 22 | 11735 | 0.06 | 0.3 |

^a^Reads counts are given in millions

^b^Expressed genes are considered when read count > 5

**Figure S1. qPCR results for *HBA* and *HBB* gene expression comparing three different globin reduction methods.**


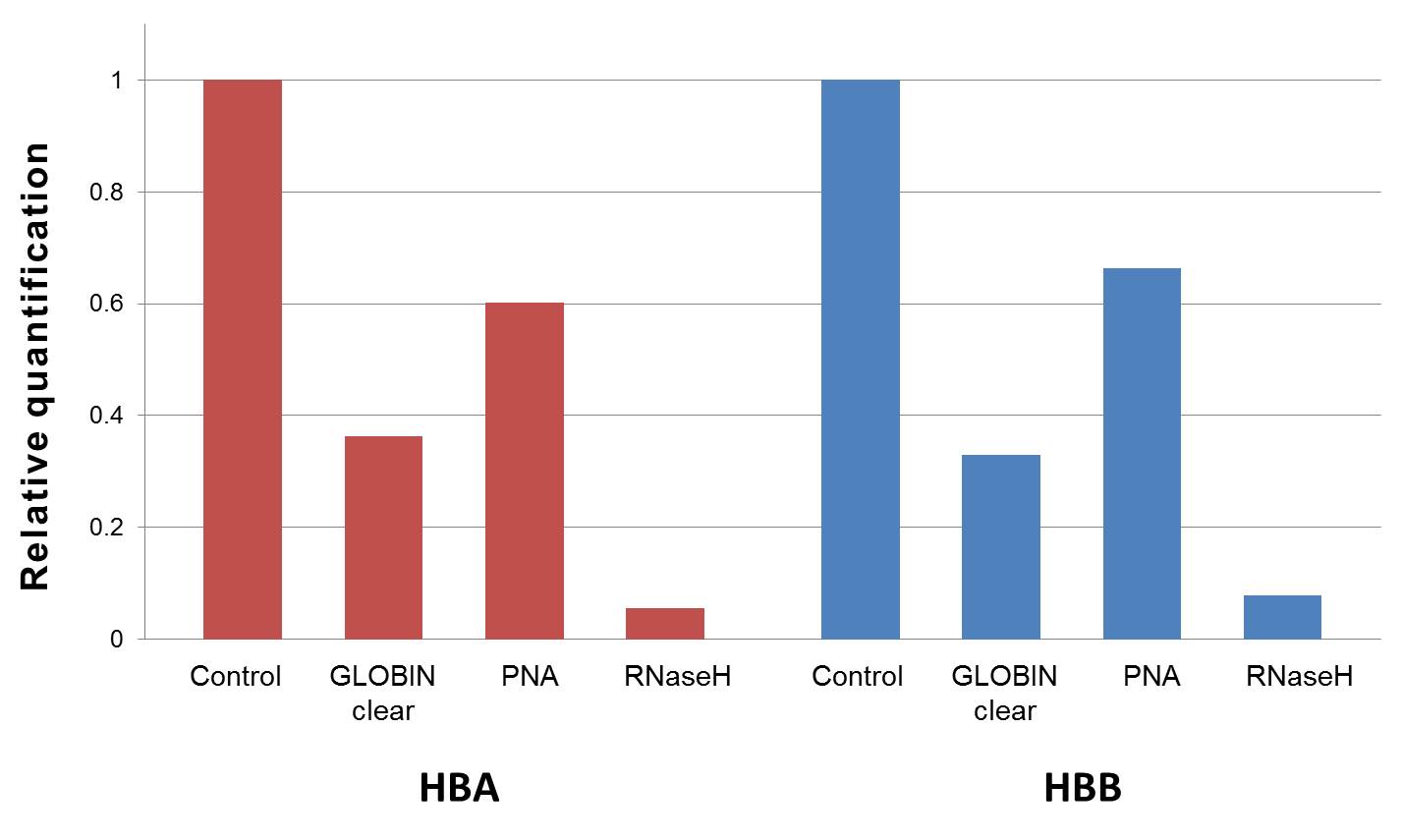


**Figure S2. Alignment of orthologous *HBA* and *HBB* cDNA sequences in human, mouse, cattle and pig.** Boxes on human sequences indicates target region hybridized with oligonucleotides in GeneChip Globin Reduction kit (Affymetrix) and boxes on capitalized pig sequences indicates target region hybridized with pig specific oligonucleotides. a) Hemoglobin, alpha (*HBA*) b) Hemoglobin, beta (*HBB*).

1. Hemoglobin, alpha (*HBA*)


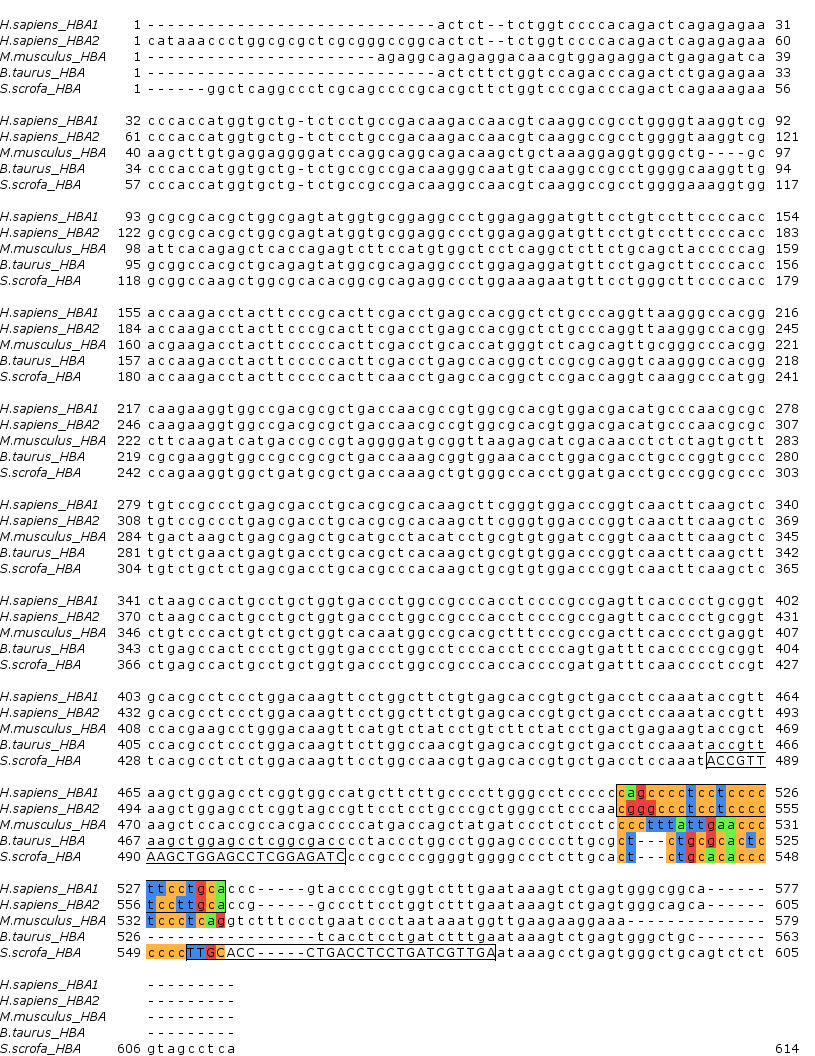


* *HBA* sequences are as follows. Human: *HBA1* ENST00000320868, *HBA2* ENST00000251595, Mouse: ENSMUST00000145569, Cattle: ENSBTAT00000037545, Pig: ENSSSCT00000008741.

b) Hemoglobin, beta (*HBB*)


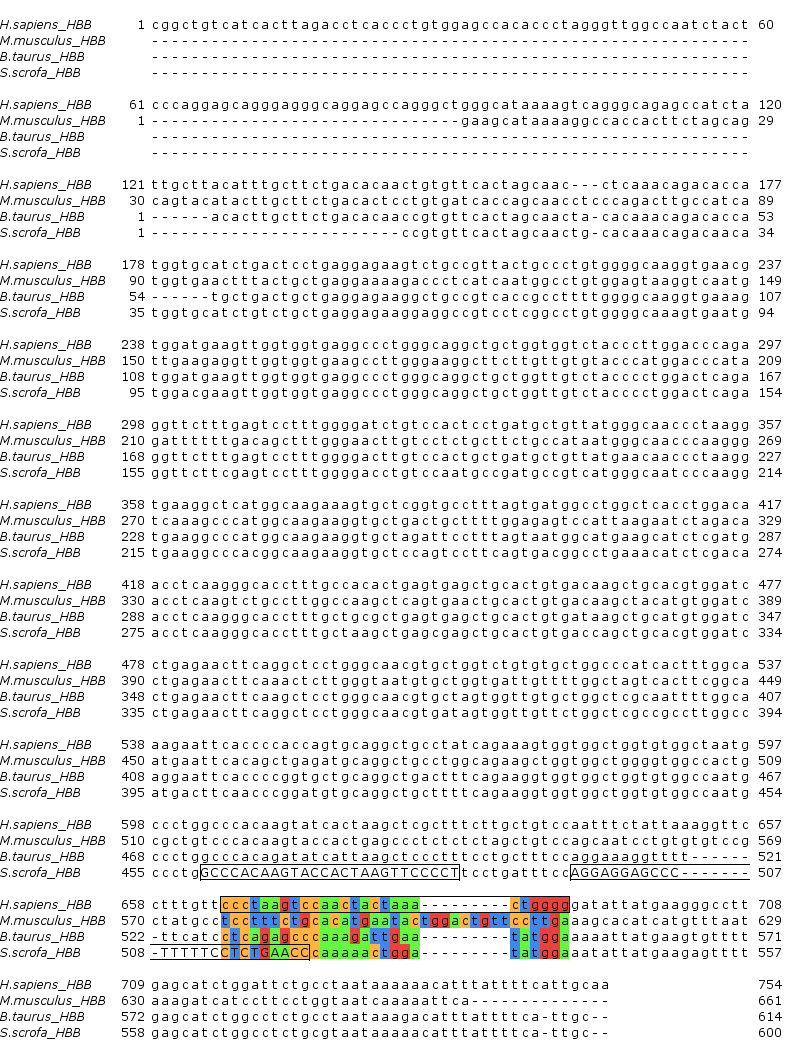


* *HBB* sequences are as follows. Human: ENST00000335295, Mouse: ENSMUST00000033229, Cattle: ENSBTAT00000045694, Pig: ENSSSCT00000036536

**Figure S3. Differential gene expression in pre- and post-GR samples.**


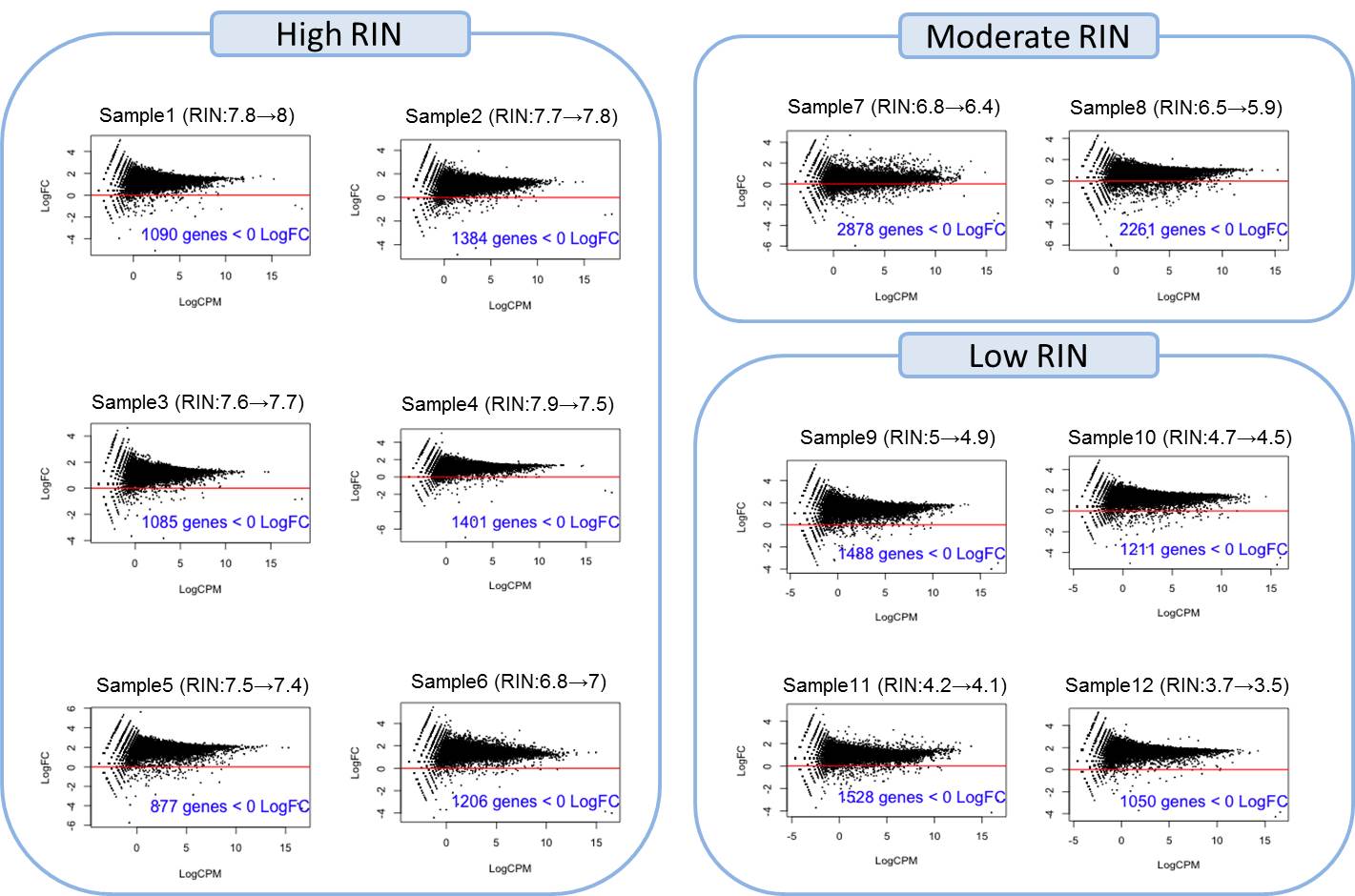


**Figure S4. Individual Venn diagrams showing the number of genes detected by RNA-seq in pre- and post-GR samples.**


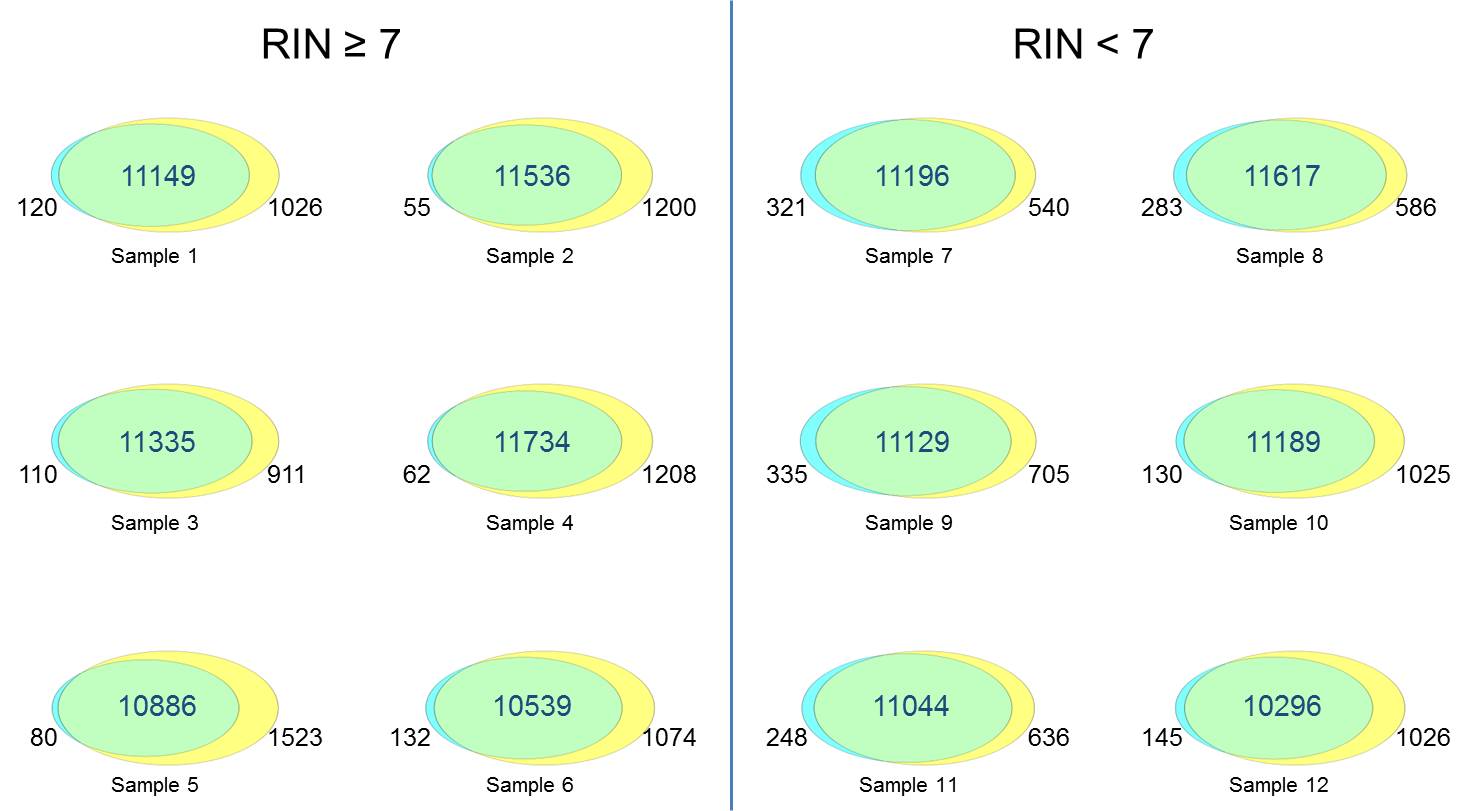


**Pre-GR**

**Post-GR**

**Post-GR**

**Pre-GR**

**Figure S5. Comparison of the mean expressions of the set of genes detected in both pre- and post-GR samples and the genes detected only in GR samples.** The mean expressions of the set of 11,773 genes detected in both pre- and post- GR samples (overlap) and the 815 genes detected only in GR samples (unique) reveals increased expression in post-GR samples.

**
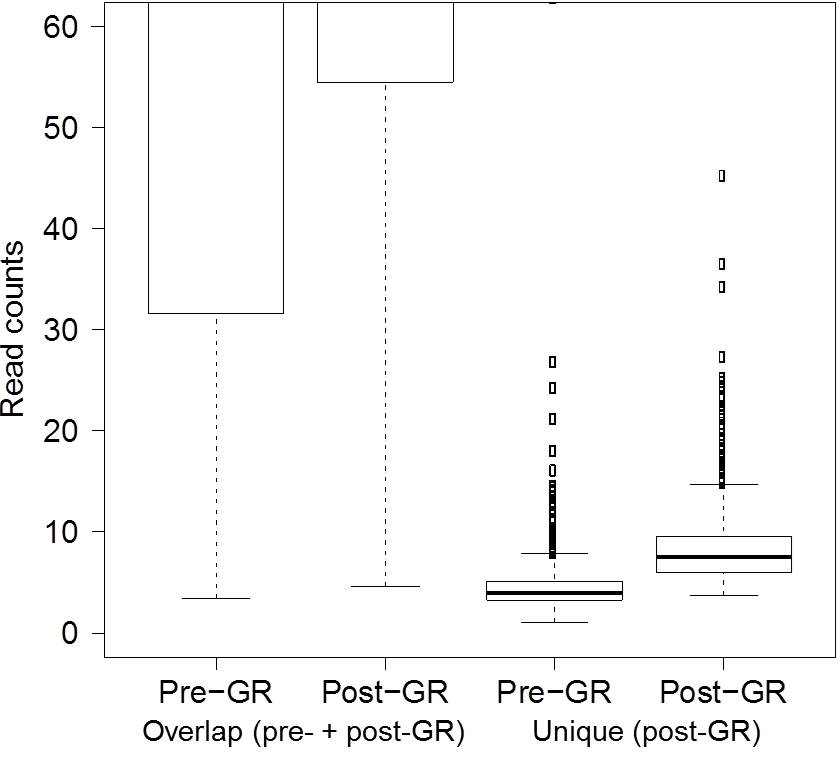
**
